# Construction of a Structurally Unbiased Brain Template with High Image Quality from MRI Scans of Saudi Adult Females

**DOI:** 10.3390/bioengineering12070722

**Published:** 2025-06-30

**Authors:** Noura Althobaiti, Kawthar Moria, Lamiaa Elrefaei, Jamaan Alghamdi, Haythum Tayeb

**Affiliations:** 1 Department of Computer Science, College of Computing and Information Technology, King Abdulaziz University, Jeddah 21589, Saudi Arabia; kmoria@kau.edu.sa; 2 Department of Electrical Engineering, Faculty of Engineering at Shoubra, Benha University, Benha 13511, Egypt; lamia.alrefaai@feng.bu.edu.eg; 3 Department of Diagnostic Radiology, Faculty of Applied Medical Sciences, King Abdulaziz University, Jeddah 21589, Saudi Arabia; jalghamdi@kau.edu.sa; 4 The Neuroscience Research Unit, Faculty of Medicine, King Abdulaziz University, Jeddah 21589, Saudi Arabia; hostayeb@kau.edu.sa

**Keywords:** Saudi brain template, population-specific template, template construction, symmetric model construction, patch-based estimation, anatomical template, structural template, MRI brain scans, T1-weighted MRI, neuroimaging

## Abstract

In brain mapping, structural templates derived from population-specific MRI scans are essential for normalizing individual brains into a common space. This normalization facilitates accurate group comparisons and statistical analyses. Although templates have been developed for various populations, none currently exist for the Saudi population. To our knowledge, this work introduces the first structural brain template constructed and evaluated from a homogeneous subset of T1-weighted MRI scans of 11 healthy Saudi female subjects aged 25 to 30. Our approach combines the symmetric model construction (SMC) method with a covariance-based weighting scheme to mitigate bias caused by over-represented anatomical features. To enhance the quality of the template, we employ a patch-based mean-shift intensity estimation method that improves image sharpness, contrast, and robustness to outliers. Additionally, we implement computational optimizations, including parallelization and vectorized operations, to increase processing efficiency. The resulting template exhibits high image quality, characterized by enhanced sharpness, improved tissue contrast, reduced sensitivity to outliers, and minimized anatomical bias. This Saudi-specific brain template addresses a critical gap in neuroimaging resources and lays a reliable foundation for future studies on brain structure and function in this population.

## 1. Introduction

Scientists have been studying the brain and nervous system for many years. Efforts to understand neuroscience contribute to improved health, save lives, and lower medical costs. Despite significant progress, there is still much that remains unknown about the brain [1] (pp. 4–21). Neuroscience is divided into various subfields to facilitate the study of the brain. One important area is neuroimaging, which employs different imaging techniques to explore the structure and function of the nervous system in a non-invasive manner. Each technique reveals distinct aspects of the nervous system [2] (pp. 459–469).

Neuroimaging techniques include structural and functional imaging. Structural imaging provides a visual representation of the brain’s anatomy, helping doctors and researchers examine brain tissues, fluids, fat, lesions, and more. Common examples of structural imaging include Computed Tomography (CT) and Magnetic Resonance Imaging (MRI). MRI can be utilized in various forms, such as T1-weighted, T2-weighted, Proton Density (PD), Fluid-Attenuated Inversion Recovery (FLAIR), Diffusion-Weighted Imaging (DWI), and Diffusion Tensor Imaging (DTI) [3] (pp. 411–417). In contrast, functional imaging reveals how the brain operates. It tracks brain activity, blood flow, metabolism, and other changes that occur in response to specific tasks or during periods of rest. Common examples of functional imaging include Functional Magnetic Resonance Imaging (fMRI), Positron Emission Tomography (PET), and Single-Photon Emission Computed Tomography (SPECT) [4] (pp. 486–488).

Neuroimaging techniques are employed in a process called brain mapping, which aids in studying the structure and function of the nervous system. Brain mapping is analogous to creating geographical maps. Just as a map of a city helps us understand its layout and organization, a brain map allows us to comprehend the arrangement of the brain. To create these maps, scientists utilize systems such as coordinate frameworks, naming hierarchies, and various imaging modalities to represent the brain from different perspectives. One of the primary objectives of brain mapping is to establish standard templates that define the outlines of different brain regions [5].

**Figure 1:**
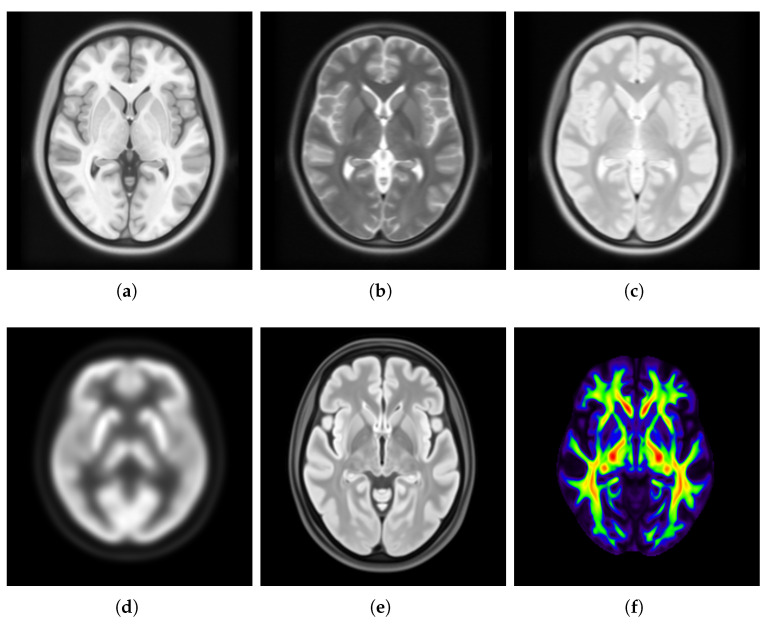
Visualization of brain templates created from various imaging modalities: (**a**) T1-weighted MRI, (**b**) T2-weighted MRI, (**c**) PD, (**d**) PET, (**e**) FLAIR, and (**f**) DTI. The grayscale contrast in (**a**–**e**) reflects the intrinsic contrast characteristics of the respective imaging modality. The colors in (**f**) represent diffusion directionality, visualized as a color-coded map.

Brain templates provide a standardized three-dimensional (3D) framework for analyzing brain data. These templates are constructed from one or more individual brains and can represent both structural and functional characteristics. By creating templates from a group of brains, researchers can uncover details that may be obscured in a single brain due to noise or individual variability [6]. These templates serve as a common reference space, allowing researchers to spatially normalize individual scans for group comparisons and statistical analyses [6,7]. Additionally, they facilitate brain tissue segmentation and the labeling of regions of interest. Figure 1 illustrates examples of brain templates created from various imaging modalities, including T1- and T2-weighted MRI, PD [8], PET [9], FLAIR, and DTI [10].

One of the earliest brain templates was the Talairach and Tournoux atlas, created in 1988. This atlas was based on a set of hand-drawn images of the right hemisphere derived from postmortem sections of a 60-year-old French female’s brain [11]. While it played a foundational role in brain mapping, its limitations—such as being based on a single brain and lacking digital precision—reduced its generalizability.

One of the first widely adopted digital brain templates was developed by the Montreal Neurological Institute (MNI) in 1993, identified as MNI-305. This template was constructed by averaging MRI scans from 305 young, healthy, right-handed Caucasian subjects [12]. Following the development of the MNI-305, the International Consortium for Brain Mapping (ICBM) developed the ICBM-152 template in 2001, using MRI scans from 152 Caucasian adults [13]. In 2003, the ICBM-452 template was introduced, created from a larger and more ethnically diverse sample, which improved its signal-to-noise ratio (SNR) [14]. However, since the majority of the subjects were Caucasian, there are ongoing concerns about the generalizability of these templates to non-Western populations.

To address the limitations of general-purpose brain templates, several population-specific templates have been developed to enhance the accuracy of brain mapping. For instance, the Chinese56 template was created in 2010 using scans from 56 young Chinese males. This template exhibited morphological differences compared with the ICBM-152 template, resulting in reduced deformation during registration [15]. Similarly, the Indian-157 template was established in 2018 from scans of 157 Indian participants and demonstrated better alignment for Indian scans compared with population-mismatched templates [16]. Other noteworthy templates include BRAHMA, which is based on T1- and T2-weighted MRI and FLAIR scans from 113 Indian subjects [17] and showed accurate segmentation. In 2020, two additional templates were introduced: one based on Caucasian data (US200) and another based on Chinese data (CN200). Both templates showed improved tissue segmentation and registration accuracy when used with population-matched scans [18]. Furthermore, the Chinese-PET templates were developed in 2021 using 116 PET scans of healthy Chinese participants. This template enhanced brain function analysis by minimizing deformation during registration [19].

Templates have become increasingly representative of specific populations when considering factors such as gender and age. The more tailored the population, the more representative the template is. For example, the Indian brain template (IBA100) takes into account both gender and nationality [20]. Several templates also incorporate age along with nationality. Notable examples include the Chinese-2020 template [21], the Chinese-children template [22], the Korean Normal Elderly template (KNE96) [23], the Indian Brain Template (IBT) [24], the Oxford-MultiModal-1 (OMM-1) template [25], the Chinese-babies template [26], and the preterm and term-born brain templates [27]. Additionally, some templates take into account nationality, gender, and age during their construction. Examples include the Korean template [28], the Chinese-1000 template [29], and the Chinese-pediatric template (CHN-PD) [30].

We have observed that numerous brain templates have been constructed for various populations; however, to the best of our knowledge, none have been specifically developed for the Saudi population. Therefore, the aim of this work is to construct a structural brain template for Saudis using T1-weighted MRI scans. To guide our selection of an appropriate methodological approach, we first review relevant studies from a computational perspective. This includes an analysis that supports our methodological choices, followed by a statement of our contributions, which is presented in Section 2, Related Work. Section 3, Materials and Methods, describes the experimental setup and procedures. It is organized into subsections detailing the Dataset (Section 3.1), the Preprocessing steps (Section 3.2), the Methodology for Template Construction (Section 3.3), and the Evaluation Methods (Section 3.4) that we used to assess our approach. Section 4, Results, presents our experimental findings, followed by Section 5, Discussion, which interprets these findings in relation to existing literature and highlights their implications. We also discuss the limitations of our study and propose directions for future research. Finally, Section 6, Conclusions, summarizes the key contributions of this work, and a list of symbols is provided at the end for reference.

## 2. Related Work

Numerous studies have utilized various methods to create templates, each driven by specific objectives. However, these methods share some common goals: achieving unbiasedness, ensuring high-quality images with sharpness, contrast, and robustness to outliers, and maintaining computational efficiency. Achieving unbiasedness means that the templates do not overly resemble any individual, whether in shape (structure), appearance (intensity), or both. This ensures that the template accurately represents the general population rather than being skewed toward specific individuals. Creating high-quality template images is crucial for accurate image registration, segmentation, and subsequent analysis. Key considerations include sharpness, which defines the clarity of edges and fine details, and contrast, which refers to the intensity differences between tissues. Equally important is robustness to outliers, which minimizes the impact of intensity variations caused by registration errors, normalization inaccuracies, or other factors. In the context of fusing aligned images to create a template, this robustness ensures that such variations do not disproportionately influence the resulting image. Finally, enhancing the computational efficiency of template construction is vital to improving the practicality of this process, particularly in large-scale studies. In the following section, we will review these studies, highlighting the specific techniques they employed to achieve their respective objectives. Additionally, Table 1 summarizes these studies and the techniques they utilized.

In 2003, Rueckert et al. [31] constructed unbiased templates from 25 T1-weighted MRI scans using statistical deformation models (SDMs) and non-rigid registration techniques. These methods ensured that the average anatomical representation reflected population variability. In 2004, Jongen et al. [32] developed an average brain image from 96 CT scans through a two-step process. First, they created a temporary average based on a subset of images. Then, they performed iterative registration of all images to this temporary average until convergence. Also in 2004, Joshi et al. [33] created unbiased templates from T1-weighted MRI scans of 50 subjects by iteratively minimizing the dissimilarity of both deformation and intensity between the population images and the average. In 2006, Christensen et al. [34] proposed a method that employs inverse consistent image registration to minimize correspondence errors, ultimately producing unbiased population average estimates from 22 T1-weighted MRI scans. Instead of merely averaging the intensities of population images mapped into a single reference space, they enhanced template sharpness by transforming the reference into the population space and averaging the resulting transformations. In 2008, Noblet et al. [35] introduced a symmetric non-rigid image registration method for constructing an average image template using 15 T1-weighted MRI scans. Their approach involved performing pairwise registrations and centering the resulting template by ensuring that the sum of all deformation fields equals zero. This method is both computationally and memory-efficient, as it relies exclusively on pairwise registrations and assumes that the deformation fields are invertible. In 2010, Avants et al. [36] applied symmetric group-wise normalization (SyGN) to T1-weighted MRI scans from 16 subjects to construct an optimal template that was unbiased in both shape and appearance within diffeomorphic space. Also in 2010, Coupé et al. [37] improved templates constructed using 20 T1-weighted MRI scans by enhancing robustness to outliers, alongside sharpness and contrast. They replaced the simple voxel-wise averaging method with a patch-based median intensity estimation within the minimum deformation template (MDT) algorithm [38], which better tolerates incorrect data values than mean-based approaches. The MDT algorithm, made publicly available in 2011 by Fonov et al. [38], was used to construct unbiased templates from 542 T1-, T2-, and PD-weighted MRI scans. Their iterative method, building on earlier works [39,40,41], aimed to minimize the mean squared differences in deformations and intensities between the template and the population at each iteration. To enhance sharpness and preserve anatomical detail, they incorporated the Automatic Nonlinear Image Matching and Anatomical Labeling (ANIMAL) algorithm [42]. In 2014, Zhang et al. [43] proposed the Volume-based Template Estimation (VTE) method using T1-weighted MRI scans from 42 subjects. This method is based on Bayesian estimation within a diffeomorphic random orbit model, which preserves the topology of brain structures and maintains image contrast without requiring cross-subject intensity averaging. In 2017, Yang et al. [44] addressed the issue of robustness to outliers while also improving sharpness and contrast in the construction of diffusion MRI templates using data from 20 subjects. They replaced traditional voxel-wise averaging with a patch-based mean-shift algorithm in wave-vector space, commonly referred to as q-space. The mean-shift algorithm [45] seeks the mode of the data distribution, providing a more robust alternative to conventional voxel-wise averaging. In 2018, Schuh et al. [46] employed a group-wise construction method to build unbiased templates from 275 T2-weighted MRI scans. Their approach involved global affine normalization, followed by deformable registration using the stationary velocity free-form deformation (SVFFD) algorithm. They enhanced sharpness through topology-preserving alignment, utilized fewer brain images per template, and applied a Laplacian sharpening filter as a post-processing step. Notably, their method achieved linear computational scalability, which contrasts with the quadratic scalability of other approaches. Also in 2018, Parvathaneni et al. [47] developed unbiased cortical surface templates from T1-weighted MRI scans of 41 subjects. They incorporated the covariance matrix from the feature space as prior knowledge in their weighting strategy, which effectively down-weights similar subjects. This allowed them to capture greater population variation while maintaining unbiasedness. In 2019, Dalca et al. [48] introduced a learning-based approach for constructing templates using convolutional neural networks (CNNs) trained on the MNIST dataset [49] with 11 classes from the Google QuickDraw dataset [50], and 7829 T1-weighted MRI scans. Their method leveraged shared information across these datasets to generate unbiased population templates conditioned on combinations of features such as age, gender, and disease status. They achieved sharpness by learning image representations that minimize spatial deformations. Unlike traditional iterative methods, which can be expensive, their approach learned a function to generate templates on demand without requiring manual data partitioning. In 2020, Ridwan et al. [51] constructed unbiased templates from 222 T1-weighted MRI scans using the widely adopted iterative technique as outlined by Joshi et al. [33], Fonov et al. [38], Guimond et al. [52]. They ensured template sharpness by incorporating high-quality scans and ensuring accurate spatial matching during the construction process. Also in 2020, Wang et al. [53] proposed a symmetric model construction (SMC) approach to generate unbiased templates from four synthetic images, 20 synthetic 3D volumes, and 20 T1-weighted MRI scans. By avoiding the use of an initial reference, their method directly determined the final unbiased template structures. To enhance sharpness, they eliminated the blurring effects typically introduced by mathematical averaging and minimized differences in both intensity and gradient information between the template and the population. This approach reformulated the registration challenge into a series of pairwise registration problems, reducing the computational cost to 2(N−1), where *N* is the total number of images. In 2023, Gu et al. [54] constructed templates from 646 T1-weighted MRI scans by incorporating deep learning (DL) techniques. They improved template sharpness using DL-mapping for image enhancement, employing CNNs with ResBlock modules. For computational efficiency, they utilized a fast DL-based registration method [55] to accelerate inter-subject registration during the template construction process. Finally, in 2023, Arthofer et al. [25] constructed unbiased templates from 240 multimodal MRI scans (T1, T2-FLAIR, and DTI) using an iterative unbiased approach described by Fonov et al. [38]. To avoid bias toward any initial reference, they computed an unbiased affine template by determining the mid-space across all subjects. They enhanced template sharpness and contrast by applying voxel-wise median calculations during the construction process.

Numerous studies have proposed methods for constructing templates, often aiming to achieve one or more of the following goals: unbiasedness, high image quality (including sharpness, contrast, and robustness to outliers), and computational efficiency. The SMC approach introduced by Wang et al. [53] provides a non-iterative, computationally efficient method for obtaining an unbiased structural template by leveraging the symmetry present in datasets. However, this assumption of symmetry can be problematic in real-world datasets, where population asymmetries may introduce bias. A similar bias issue was addressed by Parvathaneni et al. [47], who proposed a feature-based weighting scheme to down-weight contributions from over-represented data points when constructing cortical surface templates. This strategy inspired us to adopt a comparable weighting scheme within the SMC framework to enhance unbiasedness.

While the SMC method yields an unbiased structural template, it does not estimate the template intensities. To address this, we first align the population images to the template and then fuse their intensities to create the final template image. We observed that some studies improved template image quality through post-processing or by utilizing advanced techniques for fusing aligned population images. Previous works such as Coupé et al. [37] and Yang et al. [44] focused on enhancing template image quality (in terms of sharpness, contrast, and robustness to outliers) during the fusion of aligned population images. For instance, Coupé et al. [37] employed a patch-based median intensity estimation within the MDT algorithm [38] for T1-weighted MRI, while Yang et al. [44] applied a patch-based mean-shift algorithm in q-space to construct diffusion MRI templates. These methods inspired us to use patch-based intensity estimation, drawing on both approaches, and specifically tailored for T1-weighted MRI scans.

Furthermore, prior work by Miolane et al. [56] has highlighted the trade-off between achieving unbiasedness and preserving sharpness in a single population template. They recommended constructing multiple templates for homogeneous subgroups to mitigate this issue. In line with this recommendation, we selected a highly homogeneous subset of T1-weighted MRI scans from Saudi subjects. This choice aims to improve anatomical sharpness in the resulting template while maintaining unbiasedness.

**Table 1:** Summary of template-related studies on template construction, presenting key information regarding their publication year, datasets used, and the specific approaches employed to ensure unbiasedness, image quality, and/or computational efficiency.

Year	Study	Dataset	Unbiasedness	Image Quality			Efficiency
				Sharpness	Contrast	Robustness	
2003	Rueckert et al. [31]	25 T1 MRI scans	SDMs + non-rigid registration	–	–	–	–
2004	Jongen et al. [32]	96 CT scans	–	–	–	–	Two-step iterative average construction
2004	Joshi et al. [33]	T1 MRI scans of 50 subjects	Iterative minimization of deformation and intensity dissimilarity	–	–	–	–
2006	Christensen et al. [34]	22 T1 MRI scans	Inverse consistent image registration	Averaging reference transformations	–	–	–
2008	Noblet et al. [35]	15 T1 MRI scans	–	–	–	–	Symmetric pairwise non-rigid registration with invertible fields
2010	Avants et al. [36]	T1 MRI scans of 16 subjects	SyGN method	–	–	–	–
2010	Coupé et al. [37]	20 T1 MRI scans	MDT algorithm	Patch-based median estimation			–
2011	Fonov et al. [38]	542 T1, T2, and PD MRI scans	MDT algorithm	ANIMAL registration algorithm	–	–	–
2014	Zhang et al. [43]	T1 MRI scans of 42 subjects	–	VTE method		–	–
2017	Yang et al. [44]	Synthetic + diffusion MRI	–	Patch-based mean-shift algorithm			–
2018	Schuh et al. [46]	275 T2 MRI scans	group-wise method	Topology-preserving alignment, Laplacian sharpening	–	–	Linear scaling
2018	Parvathaneni et al. [47]	T1 MRI scans of 41 subjects	Feature-space covariance weighting	–	–	–	–
2019	Dalca et al. [48]	MNIST + QuickDraw + 7829 T1 MRI scans	Leveraging shared information	Reducing spatial deformations	–	–	Function to generate templates on demand
2020	Ridwan et al. [51]	222 T1 MRI scans	Unbiased iterative technique	High-quality scans and accurate spatial matching	–	–	–
2020	Wang et al. [53]	4 synthetic images + 20 synthetic 3D volumes + 20 T1 MRI	SMC approach	Iterative minimization of intensity/gradient dissimilarity	–	–	2(N−1)
2023	Gu et al. [54]	646 T1 MRI scans	–	DL-mapping sharpening	–	–	Fast DL-registration
2023	Arthofer et al. [25]	240 multimodal MRI scans	Unbiased iterative with mid-space affine	Voxel-wise medians		–	–

In this work, we construct what is, to our knowledge, the first structural brain template based on a homogeneous subset of T1-weighted MRI scans from Saudi females. Our contributions address existing gaps in the literature and aim to achieve an unbiased structural representation, high image quality (in terms of sharpness, contrast, and robustness to outliers), and computational efficiency. The main contributions of this work are as follows:

- New Population-Specific Template: We introduce a structural template derived from T1-weighted MRI scans of healthy Saudi female subjects aged 25 to 30. This template addresses a significant gap in the representation of the Saudi population in neuroimaging.
- Unbiased Template Structure with Weighting: We incorporate a covariance-based weighting scheme [47] into the SMC framework [53] to mitigate bias toward over-represented anatomical structures.
- High-Quality Intensity Estimation: We apply a patch-based intensity estimation approach, combining patch-based median estimation and the mean-shift algorithm, specifically tailored for T1-weighted MRI scans. This technique produces sharper templates with enhanced tissue contrast and robustness to outliers, outperforming traditional voxel-wise averaging.
- Computational Efficiency Enhancements: We enhance processing speed through the parallelization of independent tasks, which further improves the efficiency of the SMC framework. Additionally, we optimize matrix operations by using vectorization and filter out zero-intensity voxels during the patch-based intensity estimation process.

We expect this template to be a valuable resource for neuroimaging studies focused on the Saudi population. We also anticipate that integrating a weighting scheme within the SMC framework will reduce bias toward over-represented brain structures. Moreover, we expect that using the patch-based approach—combining median estimation with the mean-shift algorithm—will produce sharper templates with enhanced tissue contrast and robustness to outliers compared with traditional voxel-based averaging. Finally, we believe that a population-specific template, being more representative of the target group, will better preserve anatomical structures during registration.

## 3. Materials and Methods

**Figure 2:**
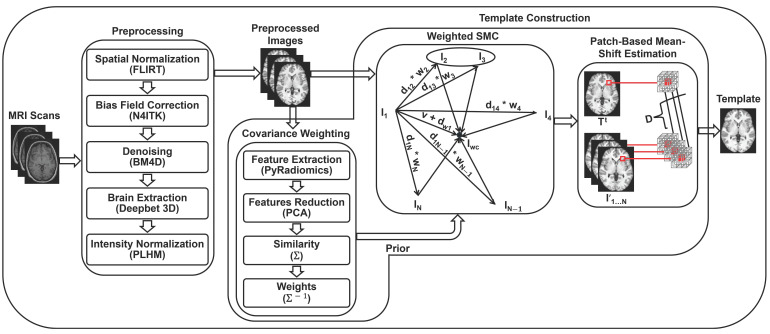
Workflow of the framework employed to construct a structural brain template for the subset of healthy Saudi adult females. It starts with the raw MRI scans on the leftmost side (data description is provided in Section 3.1), followed by preprocessing steps and the tools utilized (detailed in Section 3.2). The preprocessed images then undergo the construction process (Section 3.3). First, the similarity weights of the images are calculated using a covariance weighting scheme (Section 3.3.1). Second, these similarity weights are used as prior knowledge within the SMC framework to obtain a weighted SMC (Section 3.3.2). Notably, the incorporation of prior knowledge regarding image similarity mitigates potential bias toward similar images, such as the circled ones ($I_{2}$ and $I_{3}$). Finally, the template intensities are estimated through patch-based mean-shift estimation (Section 3.3.3), producing the final template on the rightmost side.

This section outlines the materials and methods utilized in this study. We begin by presenting the dataset employed, followed by a description of the preprocessing steps applied to it. Next, we detail the methodology used for constructing the template. Figure 2 illustrates the overall workflow, from the raw input scans to the final brain template. Finally, we describe the evaluation metrics used to assess the quality of the constructed templates.

The implementation was carried out using Google Colaboratory [57]. To enhance computational efficiency, independent processes were executed in parallel. Additionally, vectorized operations were utilized for matrix computations to further optimize performance.

### 3.1. Dataset

**Figure 3:**
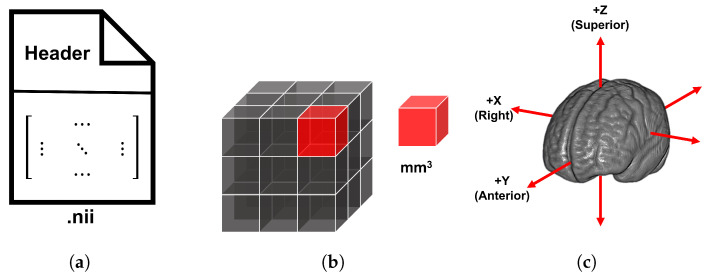
Components of a NIfTI file: (**a**) A simplified illustration of the .nii file format, which stores both metadata (in the header) and image data (voxel intensities). The image data is stored as a matrix within the file. (**b**) A visualization of the 3D image matrix composed of voxels—small cubes that represent the spatial resolution of the scan, measured in mm^3^. The small red cube represents a voxel within the shown gray matrix. (**c**) The RAS coordinate system of the scans, where the x-, y-, and z-axes, illustrated by red arrows, correspond to the Right–Left, Anterior–Posterior, and Superior–Inferior directions, respectively.

To construct and evaluate the structural brain template, we utilized a dataset consisting of 11 T1-weighted MRI scans from healthy Saudi female subjects aged 25 to 30. These scans were acquired in the Neuroimaging Informatics Technology Initiative (NIfTI) file format from King Abdulaziz University Hospital. These NIfTI files contain both a header and image data within a single file. The header stores metadata, which includes descriptive information such as file details, scanner parameters, spatial orientation, coordinate system, matrix/voxel sizes, etc. The image data consists of a matrix that stores voxel intensity values. Figure 3a illustrates a simplified representation of NIfTI file storage.

The matrix size indicates the number of voxels—small 3D cubes analogous to 2D pixels—along the x-, y-, and z-axes of the scans. Voxel size refers to the physical volume of each voxel, typically measured in cubic millimeters (mm^3^), representing the resolution of the scanned region. The scans used in this study contain 3D data matrices, as visualized in Figure 3b. Their coordinate system follows the Right–Anterior–Superior (RAS) convention, meaning the x-axis goes from right to left, the y-axis from anterior to posterior, and the z-axis from superior to inferior, as illustrated in Figure 3c.

The dataset was split into seven scans for template construction and four scans for evaluation. This dataset was selected due to its availability and the homogeneity of the subjects, which is beneficial for constructing an unbiased and sharp template [56].

### 3.2. Preprocessing

Before constructing the brain template, we preprocessed the raw MRI scans in parallel to ensure accuracy and consistency. This crucial step addressed several key challenges, including the following:

- Variability in raw data, which includes differences in matrix size, voxel size, spatial orientation, and intensity ranges.
- Scanner artifacts, such as bias fields and noise, which can affect image quality.
- Removal of irrelevant anatomical structures, such as non-brain regions, to create a brain-specific template.

To tackle these challenges, our preprocessing included several steps, each designed to standardize the data. These steps are also outlined in the pseudocode of Algorithm 1.
**Algorithm 1** Preprocessing Algorithm.1:**Input:** Raw scans $S=\{S_{1},S_{2},\ldots ,S_{N}\}:N=7$  ▹ Each scan has different image and voxel dimensions2:**Output:** Preprocessed images $I=\{I_{1},I_{2},\ldots ,I_{N}\}$      ▹ Each with image size 193×229×193 and voxel size 1×1×1 mm 3:**for** each $S_{i}\;\mathrm{in}\;S$** do**                  ▹ Processing in parallel4:   $I_{i}\leftarrow \mathrm{Spatial}\_\mathrm{Normalization}\left(S_{i}\right)$5:   $I_{i}\leftarrow \mathrm{Bias}\_\mathrm{Field}\_\mathrm{Correction}\left(I_{i}\right)$6:   $I_{i}\leftarrow \mathrm{Denoising}\left(I_{i}\right)$7:   $I_{i}\leftarrow \mathrm{Brain}\_\mathrm{Extraction}\left(I_{i}\right)$8:   $I_{i}\leftarrow \mathrm{Intensity}\_\mathrm{Normalization}\left(I_{i}\right)$9:**end for**

#### 3.2.1. Spatial Normalization

**Figure 4:**
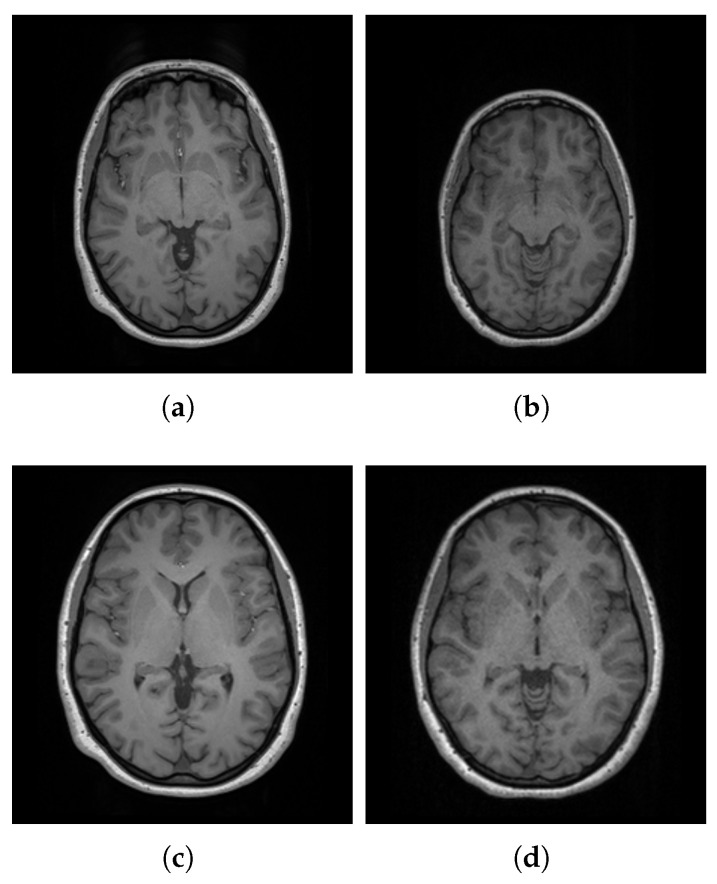
Visualization of two scans from the dataset in their native space (**a**,**b**) and after spatial normalization to the MNI152 template space (**c**,**d**).

Spatial normalization is a preprocessing step performed prior to template construction. This process involves aligning individual scans to a standard template space, effectively removing variations in brain position, orientation, size, and shape across individuals [58]. By performing this step, we ensure that the scans are comparable and exist within a similar space, as illustrated in Figure 4.

We used the updated version of MNI152 space (ICBM 2009c Nonlinear Asymmetric template) as a standard space [38,59]. We opted for the asymmetric version because it more closely resembles realistic scans. We also selected the version with ($1^{3}\;\mathrm{mm}$) resolution to enable resampling in a higher-resolution space. This standard space is archived in the TemplateFlow archive [8].

We employed affine registration, a linear but non-rigid transformation, to align the scans with the standard space. This method uses 12 degrees of freedom (DOF), which include rotation, translation, shearing, and scaling in the x, y, and z dimensions [60]. We performed the affine registration using FMRIB’s Linear Image Registration Tool (FLIRT, version 6.0) provided by FMRIB Software Library (FSL, version 6.0.5.2) [61,62,63]. The cost function we used was normalized cross-correlation, as it is well-suited for intramodality registration. After completing the affine registration, the images were resampled into the standard space using spline interpolation. Spline interpolation was chosen for its ability to accurately preserve anatomical details while providing smooth transformations. Due to the potential for spline interpolation to introduce negative values, these values were set to zero to maintain valid image intensities.

#### 3.2.2. Bias Field Correction

The magnetic field within the scanner is not uniform, which can cause artifacts in the scan that alter the intensity values. This artifact is known as bias field or intensity inhomogeneity [64]. The bias field can cause the same tissue to have different intensity values, affecting subsequent image processing [65]. Therefore, it should be corrected as a preprocessing step for constructing the brain template.

**Figure 5:**
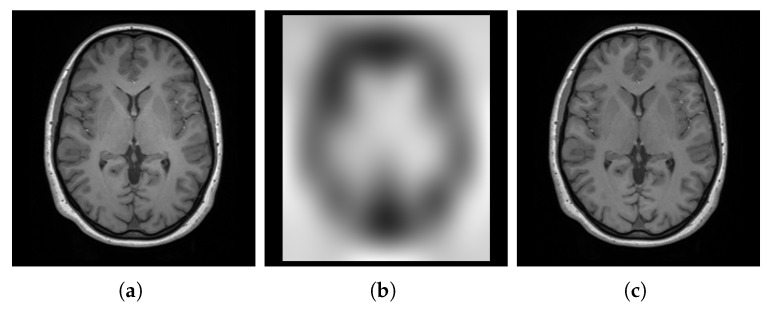
Visualization of a spatially normalized image corrupted by a bias field (**a**). (**b**) shows the estimated bias field. (**c**) displays the image after correction using the N4 algorithm.

We corrected the bias field by applying the N4 algorithm [66] using the Simple Insight Toolkit (SimpleITK, version 2.4.0) [67]. The N4 algorithm assumes that the corrupted image combines the true underlying image and the bias field, with negligible additional noise. It estimates these merged parts iteratively using a hierarchical optimization scheme (i.e., a multi-resolution scheme) where the image is processed at increasing levels of resolution. This iterative process effectively estimates and corrects for the bias field. Figure 5 shows an image, from the spatially normalized images, before and after using N4 and the estimated bias field.

#### 3.2.3. Denoising

Noise in MRI scans is a random variable that contributes to the detected signal. This noise arises from various sources, including thermal noise in the scanner and the lossy interactions between the scanner and the scanned body [68]. Denoising is essential to improve the SNR, which can significantly impact subsequent image processing, analyses, and quantitative measurements [69,70]. Accurate denoising is crucial for template construction, as it ensures that the template reflects true anatomical features rather than noise artifacts.

**Figure 6:**
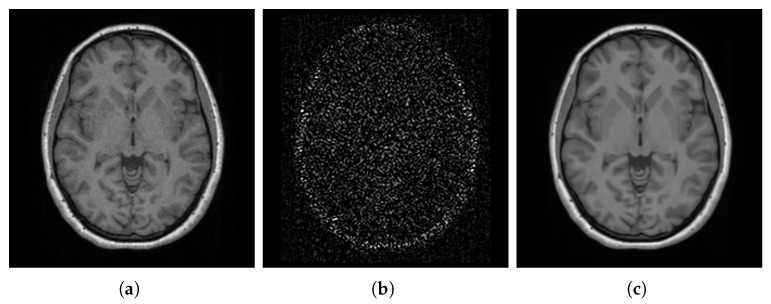
Visualization of a noisy image (one of the bias field corrected images) (**a**), estimated noise (**b**), and denoised image (**c**).

We applied the block-matching and 4D filtering (BM4D, version 4.2.4) algorithm to denoise our images. BM4D is a powerful denoising technique that exploits local and nonlocal correlations between voxels to effectively separate signal and noise while preserving sharp edges [71,72,73]. The BM4D algorithm requires an initial estimation of the noise standard deviation (SD). We estimated the noise SD from the image background where no anatomical structures were present. Figure 6 visualizes an image, from the bias field corrected images, before and after the process of denoising, along with the estimated noise.

#### 3.2.4. Brain Extraction

Brain extraction, also known as skull stripping, is the process of separating the brain from the non-brain regions, reducing unwanted information that could interfere with subsequent processes. This essential preprocessing step facilitates various image processing tasks for the brain region, including intensity normalization, registration, template construction, and tissue segmentation [74,75,76]. However, brain extraction can be skipped when constructing head templates.

**Figure 7:**
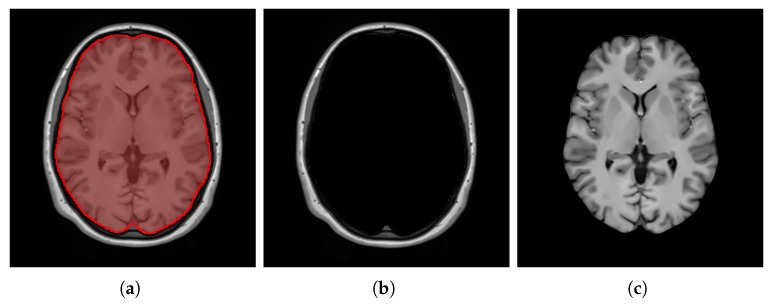
Visualization of the whole head with the estimated brain mask overlay of one of the denoised images (**a**), the excluded non-brain regions (**b**), and the extracted brain (**c**).

To extract the brains, we applied a fast and high-resolution method called deepbet 3D (version 1.0.2). This DL-based method was trained on 568 T1-weighted MRI scans of healthy adults and utilizes LinkNet [77], a modern architecture built upon the UNet framework [78], to perform the extraction in two stages. In the first stage, the model predicts an initial mask, which is then used to crop the MRI scan, focusing specifically on the brain region. In the second stage, the cropped MRI scan undergoes further processing to predict a more accurate final brain mask [79]. Figure 7 shows the final estimated mask overlaid on the entire head for a sample image from the denoised images, along with the excluded non-brain regions as well as the final extracted brain.

#### 3.2.5. Intensity Normalization

The intensity values of MRI scans are influenced by factors related to both the inherent properties of the tissue being scanned and scanner-related parameters [80]. MRI scan intensities, unlike typical image intensities that range from 0 to 255, start at zero and have no upper limit, as the important thing is that there is contrast between tissues, regardless of the specific intensity values. This situation can lead to inconsistent intensity interpretation across different scans. Thus, performing intensity normalization as a preprocessing step is crucial to ensure that the images are on a consistent scale, improving the quality and reliability of medical imaging processes and analyses [81,82] and the brain template construction.

**Figure 8:**
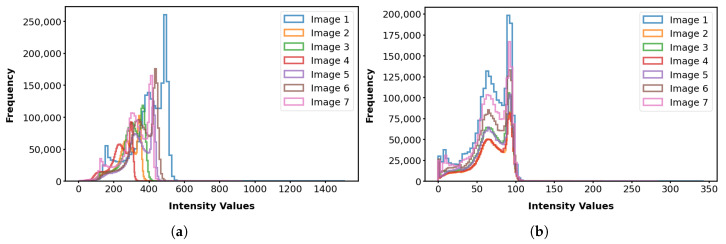
Visualization of the unnormalized intensity histograms (**a**) and the corresponding normalized intensity histograms (**b**) for the brain-extracted images.

We normalized the image intensities using the piecewise linear histogram matching (PLHM) method, which involves two main stages: training and transformation. During the training stage, standard histogram landmarks are learned from a set of images. Then, in the transformation stage, the intensity of each image is mapped to the learned standard histogram [83]. We applied the PLHM implementation wrapped in a tool named intensity-normalization (version 2.2.4) [82], where a predefined lower and upper bound of 0 and 100 is arbitrarily set for the standard histogram. Figure 8 illustrates the intensity histograms of the brain-extracted images before and after the intensity normalization process.

### 3.3. Template Construction

Our template construction methodology comprises two main parts: First, obtaining unbiased template structures using SMC [53]. SMC directly estimates the unbiased template structure without iterative optimization. We further incorporate a covariance weighting scheme, based on the work of Parvathaneni et al. [47], to account for any asymmetry in the population, ensuring that the template is not biased towards over-represented brain structures. Second, estimating the template intensity using patch-based estimation, inspired by the work of Coupé et al. [37], Yang et al. [44]. Patch-based estimation provides robustness to outliers and helps to preserve image details, leading to a high-quality template image. The details of each step are explained in the following sections, and the full procedure is outlined in the pseudocode of Algorithm 2.
**Algorithm 2** Template Construction Algorithm. 1:**Input:** Preprocessed images $I=\{I_{1},I_{2},\ldots ,I_{N}\}$    ▹ Each with image size 193×229×193 and voxel size 1×1×1 mm 2:**Output:** Template *T*         ▹ With image size 193×229×193 and voxel size 1×1×1 mm  3:**Step 1: Covariance Weighting** 4:   F←Extract_Features(I)                     ▹ Processing in parallel, N×107 5:   PC←PCA(F)                                 ▹N×5 6:   Σ←Covariance(PC)                              ▹N×N 7:   $W\leftarrow \mathrm{Row}\_\mathrm{Wise}\_\mathrm{Summation}(\Sigma^{-1})$                       ▹N×1 8:   $NW\leftarrow \frac{W}{\sum W}$                                 ▹N×1  9:**Step 2: Weighted SMC**10:   $I_{j}\leftarrow \mathrm{any}\;\mathrm{image}\;\mathrm{from}\;I$11:   **for** each $I_{i}\;\mathrm{in}\;I:i\neq j$** do**                     ▹ Processing in parallel12:      $d_{ji}\leftarrow \mathrm{Align}\left(I_{j},I_{i}\right)$                         ▹193×229×193×1×313:   **end for**14:   $d_{wj}\leftarrow \sum_{\begin{array}{c} i=1\\ i\neq j \end{array}}^{N}d_{ji}*NW_{i}$                        ▹193×229×193×1×315:   $I_{wc}\leftarrow I_{j}\left(v+d_{wj}\right)$                             ▹193×229×19316:   **for** each $I_{i}\;\mathrm{in}\;I:i\neq j$** do**                     ▹ Processing in parallel17:      $I_{i}^{\prime }\leftarrow \mathrm{Align}\left(I_{i},I_{wc}\right)$                           ▹193×229×19318:   **end for** 19:**Step 3: Patch-Based Mean-Shift Estimation**                 ▹ Vectorized20:   $T^{0}\leftarrow \mathrm{Median}\left(I^{\prime }\right)$               ▹ Initialize template of size 193×229×19321:   t←1                          ▹ Initialize iteration counter22:   max_itr←200                    ▹ Maximum number of iterations23:   $V\leftarrow \{v|T^{0}\left(v\right)\neq 0\}$                    ▹ Set of *K* nonzero voxel indices24:   **while** True **do**25:      $P_{T}\leftarrow \mathrm{Extract}\_\mathrm{Patches}\left(T^{t-1},V\right)$                        ▹K×3×3×326:      $P_{I}\leftarrow \mathrm{Extract}\_\mathrm{Patches}\left(I^{\prime },V\right)$                        ▹N×K×3×3×327:      $D\leftarrow ∥P_{T}-P_{I}{∥}_{2}$                                ▹N×K28:      h←Median(D)                                ▹1×K29:      $w\leftarrow \exp\left(-\frac{D^{2}}{h}\right)$                               ▹N×K30:      $nw\leftarrow \frac{w}{\sum w}$                                  ▹N×K31:      $T^{t}\left(V\right)\leftarrow \sum_{i=1}^{N}nw_{i}*I^{\prime }\left(V\right)$                        ▹193×229×19332:      $\Delta \leftarrow ∥T^{t}-T^{t-1}{∥}_{2}$33:      **if** $\Delta <10^{-6}$** or **t=max_itr **then **34:         $T\leftarrow T^{t}$                          ▹ The final template values35:         **break**36:      **else**37:         t←t+1                        ▹ Update the iterations counter38:      **end if**39:   **end while**

#### 3.3.1. Covariance Weighting

This step adapts the approach described by Parvathaneni et al. [47] for constructing unbiased cortical surface templates. The core idea is to deweight similar data points to maximize the captured variance within the population. We began by extracting 107 radiomic features (*F*) from each preprocessed image (*I*) in parallel using the PyRadiomics Python package (version 3.0.1) [84]. These features, encompassing First Order Statistics, Shape, and Texture characteristics, were extracted in segment-based mode, yielding a single value per feature for the brain region. This comprehensive set of features provides a quantitative representation of the image data.

To reduce the dimensionality of the feature space and mitigate potential issues with multicollinearity, we applied Principal Component Analysis (PCA) [85] to the extracted features (*F*). We retained the top five principal components (PC) which captured 95% of the total variance in the data. We then calculated the covariance matrix (Σ) of these principal components. Next, we computed the pseudo-inverse (Moore–Penrose inverse) [86] of the covariance matrix, denoted as $\Sigma^{-1}$.

For each image ($I_{i}$), we calculated its weight ($W_{i}$) by summing the elements in the $i^{th}$ row of $\Sigma^{-1}$. This sum reflects the image’s dissimilarity to the rest of the population; a larger sum indicates greater dissimilarity. We then normalized these weights by dividing each weight by the sum of all weights, resulting in normalized weights NW that sum to one. This normalization ensures that the weights can be interpreted as proportions and is useful for subsequent steps. Table 2 presents the calculated similarity weight for each image. Higher weights indicate that an image is more distinct from the rest of the population, while lower weights suggest greater similarity.

#### 3.3.2. Weighted SMC

This step adapts the SMC method proposed by Wang et al. [53] for unbiased template construction. The SMC method assumes that any image in the population can reach the population center directly without iterative averaging, thereby improving efficiency. However, when applied to potentially asymmetric population images, this direct approach can be sensitive to biases introduced by groups of similar images. To address this, we incorporate the similarity weights derived in the previous step to guide the center calculation and mitigate potential biases.

The process begins by selecting any image ($I_{j}$) from the preprocessed images (*I*). Next, we align or register $I_{j}$ to each $I_{i}$ in *I* in parallel, such that i≠j, to obtain the displacements ($d_{ji}$). We performed this alignment using the Symmetric Diffeomorphic Normalization (SyN) algorithm from the Advanced Normalization Tools in Python (ANTsPy, version 0.5.4) [87]. SyN is a robust nonlinear registration algorithm known for its ability to handle complex deformations while preserving anatomical topology [88,89,90].

To account for the varying similarity of images in the population, we calculate a weighted displacement ($d_{wj}$) that incorporates the normalized weights (NW) obtained in the previous step (Section 3.3.1). Specifically, $d_{wj}$ is calculated as the weighted sum of the individual displacements:

(1) $$d_{wj}\leftarrow \sum_{\begin{array}{c} i=1\\ i\neq j \end{array}}^{N}d_{ji}*NW_{i}.$$

**Figure 9:**
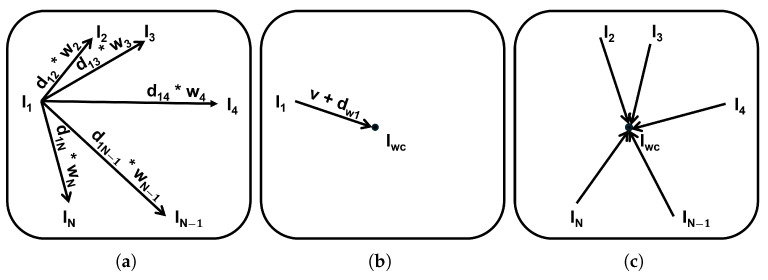
Illustration of the weighted SMC: (**a**) shows the weighted individual displacements, where the asterisk (*) denotes element-wise multiplication. These weighted displacements are then summed and applied to $I_{1}$ to estimate the population center $I_{wc}$ (**b**). The final step involves aligning all images to this estimated center (**c**). Note that this illustration is adapted from Wang et al. [53].

The weighted displacement is then applied to $I_{j}$ using the ApplyTransforms function of the ANTsPy [87], resulting in $I_{j}\left(v+d_{wj}\right)$, where *v* denotes voxel indices. This yields the moved image $I_{j}^{\prime }\left(v\right)$, which represents the weighted center ($I_{wc}$) of the set *I*. Finally, all remaining $I_{i}$ in *I* are aligned to $I_{wc}$ in parallel using SyN registration to facilitate further processing of the template’s voxel intensities in Section 3.3.3. This weighted SMC step is also visualized in Figure 9 for further illustration.

**Figure 10:**
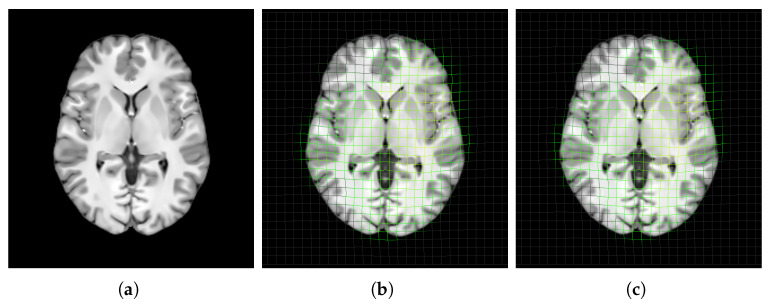
Visualization of the center reached by applying displacement, illustrated as a grid overlay, to a random image shown in (**a**), once with similarity weights (**b**) and once without (**c**).

**Figure 11:**
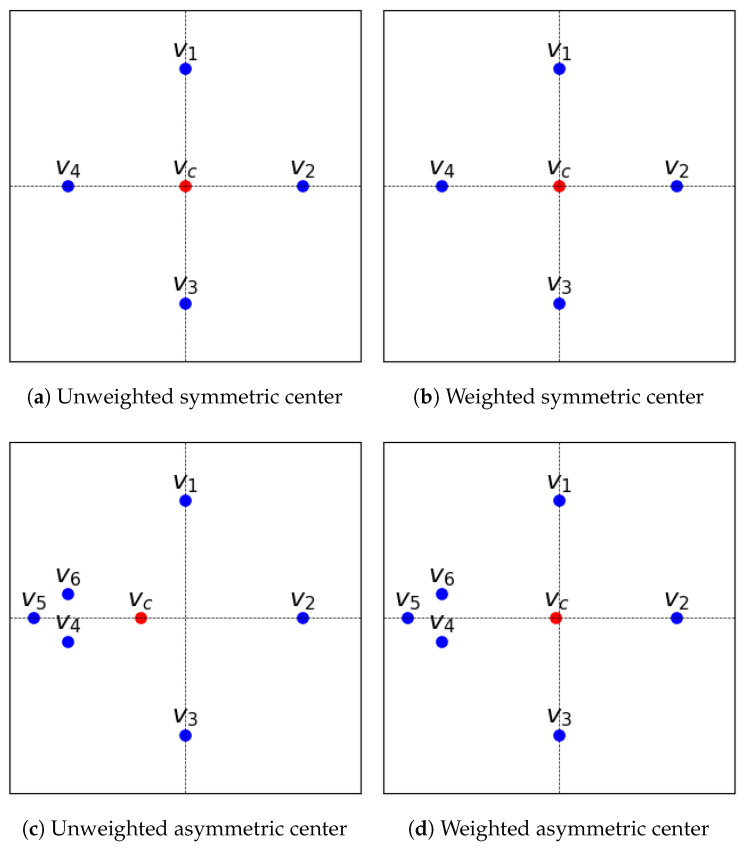
Comparison of symmetric and asymmetric center ($v_{c}$) computation for voxel locations (*v*) with and without similarity weight knowledge: (**a**,**c**) show the unweighted symmetric and asymmetric center, respectively, computed without similarity weights, where in (**c**) $v_{c}$ is biased towards the similar subset $v_{4}$, $v_{5}$, and $v_{6}$. (**b**,**d**) show the weighted symmetric and asymmetric center, respectively, incorporating similarity weights, which reduces the bias towards the similar subset observed in (**c**). Note that this toy example is adapted from Parvathaneni et al. [47].

Figure 10 visualizes the image $I_{j}$, along with the weighted displacement ($d_{wj}$) and the weighted center ($I_{wc}$), as well as the displacement ($d_{j}$) and the center ($I_{c}$) without weighting, which was calculated for further evaluation in Section 3.4.1. In Figure 11, we visualize a toy example on a 2D plane to illustrate the incorporation of similarity weights as prior knowledge to the center ($v_{c}$) computation. When the voxel locations (*v*) are distributed symmetrically, they receive similar weights. Therefore, using the weight information to compute $v_{c}$ has no effect on the result. However, when the *v* are distributed asymmetrically, they receive different weights. In this case, incorporating the weight knowledge reduces the bias of $v_{c}$ towards the similar subset.

#### 3.3.3. Patch-Based Mean-Shift Estimation

To obtain the final template, we fuse the intensities of the aligned population images ($I^{\prime }$) using a robust and adaptive patch-based estimation scheme. This approach addresses the limitations of the voxel-based simple averaging method, which is susceptible to blurring and the influence of outliers that may arise from imperfect image alignment. Our method builds upon the work of Coupé et al. [37], Yang et al. [44], which demonstrated the advantages of patch-based estimation [91] for constructing sharp and robust templates. They replaced the voxel-based simple averaging method with a patch-based median estimation [37] and patch-based mean-shift algorithm [44]. Here, the median can tolerate incorrect data values, and the mean-shift algorithm [45] seeks the mode of the data distribution, providing a more robust alternative to the voxel-based simple averaging method.

We initialize the template $T^{0}$ using the median intensities of the aligned images ($I^{\prime }$). To improve the efficiency of the iterative estimation, we leverage matrix vectorization and restrict computations to nonzero voxels only. We define a set of nonzero voxel indices (*V*) of length *K*. For each voxel index *v* in *V*, we extract a 3×3×3 patch centered at *v* from both the template ($P_{T}$) and each aligned image in the set $I^{\prime }$ ($P_{I}$).

**Figure 12:**
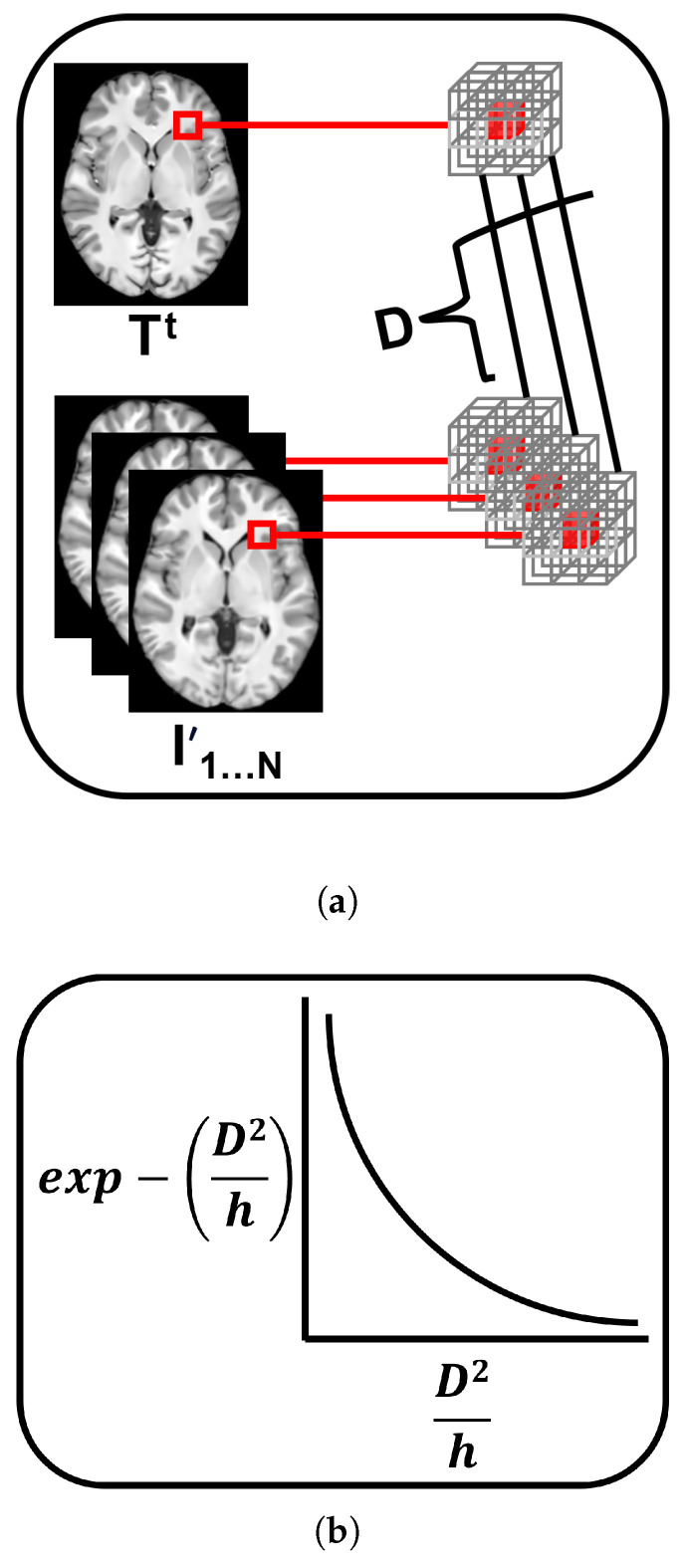
Illustration of the computed distances *D* between the template patch and each corresponding patch in the aligned images (**a**), where the patches are represented as gray 3D matrices surrounding a voxel, shown as a small red cube. Panel (**b**) shows the exponential function used to compute the weights *w*, where *h* is the median of the distances *D* and serves as a dynamic parameter controlling the decay rate of the function.

Next, we compute the Euclidean distances (*D*) between the template patch and each corresponding patch in the aligned images. These distances are then used to compute weights *w* using a Gaussian kernel:

(2) $$w\leftarrow \exp\left(-\frac{D^{2}}{h}\right),$$

where *h*, the median of the distances *D*, serves as a dynamic Gaussian bandwidth parameter. This parameter controls the decay of the exponential function, thereby affecting how each image’s voxel intensity influences the template update. Figure 12 illustrates the computed *D* between the template patch and each corresponding patch in the aligned images, alongside the function used to compute *w*.

The weights are then normalized to sum to one, producing normalized weights (nw). These weights are used to compute a weighted average of the voxel intensities across the aligned images. At each iteration *t*, the template’s nonzero voxel intensities are updated as follows:

(3) $$T^{t}\left(V\right)\leftarrow \sum_{i=1}^{N}nw_{i}*I^{\prime }\left(V\right).$$

where $I_{i}^{\prime }\left(V\right)$ represents the intensity values of the $i^{\mathrm{th}}$ aligned image at the nonzero voxel indices.

**Figure 13:**
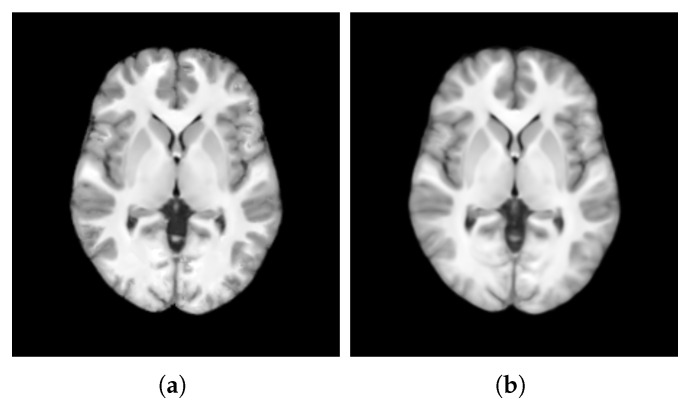
Visualization of the templates obtained from patch-based estimation (**a**) and voxel-based averaging (**b**).

This process is repeated until the difference between successive templates (Δ) is less than $10^{-6}$, or until the maximum number of iterations is reached. Figure 13 visualizes the final template obtained from this patch-based estimation ($T_{P}$), alongside a template generated using voxel-based simple averaging ($T_{V}$), which is further evaluated in Section 3.4.2.

### 3.4. Evaluation Methods

Our evaluation of the constructed templates focuses on three aspects:

- Evaluating the structural unbiasedness of the templates computed in Section 3.3.2.
- Assessing the intensity quality of the templates computed in Section 3.3.3, in terms of sharpness, contrast, and robustness to outliers.
- Investigating the necessity of constructing a brain template specifically for Saudi adult females by evaluating its effectiveness as a target registration space in comparison with other population-specific templates.

#### 3.4.1. Unbiasedness of Template Structure

To assess the impact of similarity weights on the unbiasedness of the computed centers, we compared the results obtained using similarity weights ($I_{wc}$) with those obtained without ($I_{c}$), as detailed in Section 3.3.2. For each image in the population, we computed the squared magnitude of its displacement from the corresponding center image ($I_{WC}$ or $I_{C}$). The displacement was obtained using the SyN registration of the ANTsPy [87]. To weight the displacement of each image according to its similarity within the population, we incorporated the similarity weight (NW) computed in Section 3.3.1. This metric, which we refer to as weighted displacement (WD)—adapted from Wang et al. [53]—quantifies the degree of bias in the computed center; lower WD values indicate less bias:

(4) $$WD=\sum_{i=1}^{N}{∥d_{ic}∥}_{2}^{2}*NW_{i},$$

where *N* is the total number of images in the population, $d_{ic}$ is the displacement from image $I_{i}$ to the center image $I_{WC}$ or $I_{C}$, ${∥.∥}_{2}^{2}$ is the squared L2-norm, and $NW_{i}$ is the similarity weight for image $I_{i}$.

#### 3.4.2. Quality of Template Intensity

In this section, we assess the quality of the templates’ intensity obtained from both patch-based estimation ($T_{P}$) and voxel-based averaging ($T_{V}$) in Section 3.3.3 across three evaluation metrics.

- Sharpness
  To evaluate the sharpness and edge definition of the templates, we computed the magnitude of the gradient for each voxel. Sharp edges and well-defined details correspond to regions with rapid changes in intensity, which are reflected in high gradient magnitudes. To assess the overall sharpness, we averaged the gradient magnitudes across all voxels in the template. This metric—as used in Wang et al. [53]—which we refer to as Average Gradient Magnitude (AGM), quantifies the overall sharpness of the template:
  (5) $$AGM=\frac{1}{M}\sum_{v}{∥\nabla T(v)∥}_{2},$$
  where T(v) is the intensity value of the template at voxel index *v*, *∇* is the gradient operator, and *M* is the total number of voxels in the template.
- Contrast
  To evaluate the contrast between white matter (WM) and gray matter (GM) of the templates, we used the Normalized Michelson Contrast [92]. This metric provides a standardized measure of contrast by comparing the maximum intensity of WM to the minimum intensity of GM. To identify WM and GM voxels, we utilized the BrainSuite tool (version 23a) [93] to segment the templates into different tissue types. This allowed us to isolate voxels corresponding to pure WM and GM, excluding those with other tissue types. It is worth noting that we utilized the BrainSuite tool [93] on a local machine, not within the Google Colaboratory environment [57]. This metric, Normalized Michelson Contrast (NMC), quantifies the contrast between WM and GM; higher values indicate greater contrast:
  (6) $$NMC=\frac{WM_{max}-GM_{min}}{WM_{max}+GM_{min}},$$
  where $WM_{max}$ is the maximum intensity value within the WM voxels, and $GM_{min}$ is the minimum intensity value within the GM voxels.
- Robustness to Outliers
  To assess the robustness to outliers of the templates, we used the Kullback-Leibler Divergence [94]. This metric, denoted as $D_{KL}$, measures the similarity between the intensity distributions of the templates and the population. We introduced an outlier image by adding noise to one of the images to make its intensity distribution significantly different. For each template, we computed the $D_{KL}$ between its intensity distribution and the intensity distribution of each image in the population. Lower $D_{KL}$ values indicate greater similarity between the two distributions, with a value of 0 indicating identical distributions:
  (7) $$D_{KL}\left(P||Q\right)=\sum_{x}P\left(x\right)\log\left(\frac{P(x)}{Q(x)}\right),$$
  where P(x) represents the probability of intensity value *x* in the template’s distribution, and Q(x) represents the probability of intensity value *x* in the population image distribution.

#### 3.4.3. Usability of Saudi Brain Template

To investigate the necessity of constructing a population-specific brain template, we compared the deformations required to nonlinearly register new healthy Saudi adult female brain images to our proposed brain template, constructed using Patch-Based Mean-Shift Estimation (Section 3.3.3), as well as to other population-based templates. For ease of reference, we refer to our template as the Brain Template for Healthy Saudi Adult Females (BT-HSAF). Since population-specific characteristics, such as ethnicity, gender, and age, can influence brain morphology, it is crucial to compare templates with similar demographic features for accurate assessment. Therefore, we focused on templates that are similar to our BT-HSAF in terms of age, gender, or both. Specifically, we utilized the Caucasian (US200), Chinese (CN200) [18], and Indian (IBA100) [20] templates. To ensure accurate comparisons by removing linear variations, we first affinely aligned all templates using the ANTsPy [87].

To assess the representativeness of each template, we used the four evaluation scans described in Section 3.1. We extracted the brains using deepbet 3D [79]. Next, we affinely aligned the extracted brains to each template to account for linear variations. Then, we nonlinearly registered each brain to each template using SyN registration [87], resulting in deformation fields representing the transformations needed to warp each brain onto each template.

To quantify the local changes in brain volume during registration, we computed the mean of the logarithm of the Jacobian determinant (mLJD)—as used in Yang et al. [18]—for each deformation field. mLJD provides information about the voxel-wise volume changes, where positive values indicate expansion and negative values indicate compression. We used the CreateJacobianDeterminantImage function of ANTsPy [87] to calculate the LJD. Values of mLJD closer to zero indicate fewer deformations, suggesting a higher similarity between the template and the population. It is calculated as follows:

(8) $$mLJD=\frac{1}{M}\sum_{v}\log(|\det\left(J_{v}\right)\left|\right),$$

where $J_{v}$ is the Jacobian matrix at voxel index *v*, $\det(J_{v})$ is the determinant of the Jacobian matrix at voxel index *v*, and the absolute value $\left|\det(J_{v})\right|$ ensures that the logarithm is defined, as the determinant can be negative.

## 4. Results

This section presents the results of evaluating three key aspects:

- The unbiasedness of the template structure computed with versus without incorporating weights.
- The quality of template intensity using patch-based estimation versus voxel-based averaging.
- The necessity of using a brain template specifically tailored to healthy Saudi adult females as the standard space for registering subjects from the same population.

### 4.1. Unbiasedness of Template Structure

Table 3 presents the sum and average of WD over all voxels for both $I_{wc}$ and $I_{c}$. The sum of WD for $I_{wc}$ (33,566,831.060) was lower than that of $I_{c}$ (33,735,950.577), as was the average ($I_{wc}$: 3.935, $I_{c}$: 3.955). The lower WD values for $I_{wc}$ indicate that incorporating similarity weights resulted in a center image with reduced bias compared with $I_{c}$. This finding suggests that weighting images based on their similarity to the population can lead to a more representative and unbiased template.

### 4.2. Quality of Template Intensity

In this section, we summarize the results of assessing the quality of the templates’ intensity obtained from both patch-based estimation ($T_{P}$) and voxel-based averaging ($T_{V}$) (visualized in Figure 13), conducted using AGM, NMC, and $D_{KL}$.

**Figure 14:**
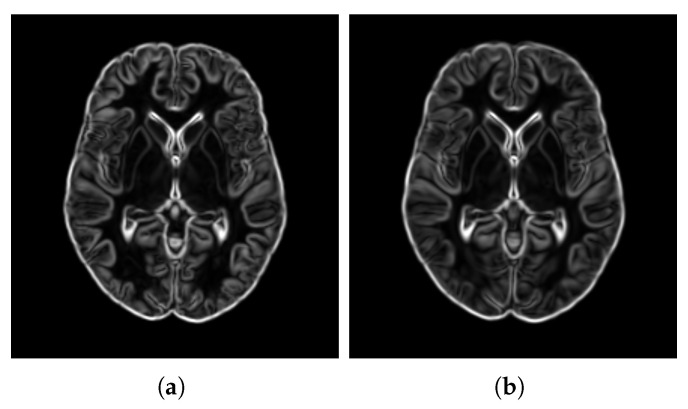
Visualization of the gradient magnitude of the templates obtained from patch-based estimation (**a**) and voxel-based averaging (**b**).

**Figure 15:**
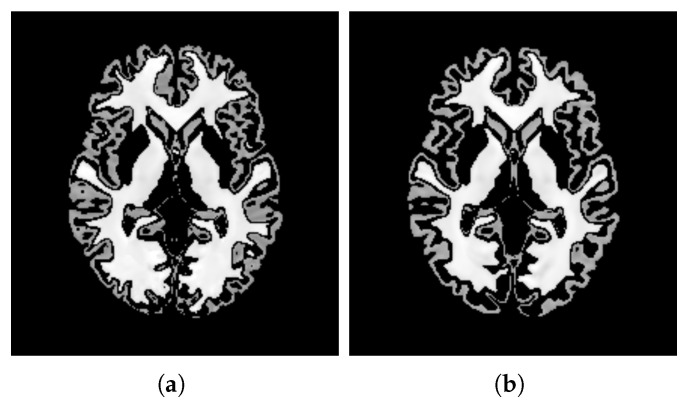
Visualization of pure WM and GM regions in templates generated using patch-based estimation (**a**) and voxel-based averaging (**b**).

**Figure 16:**
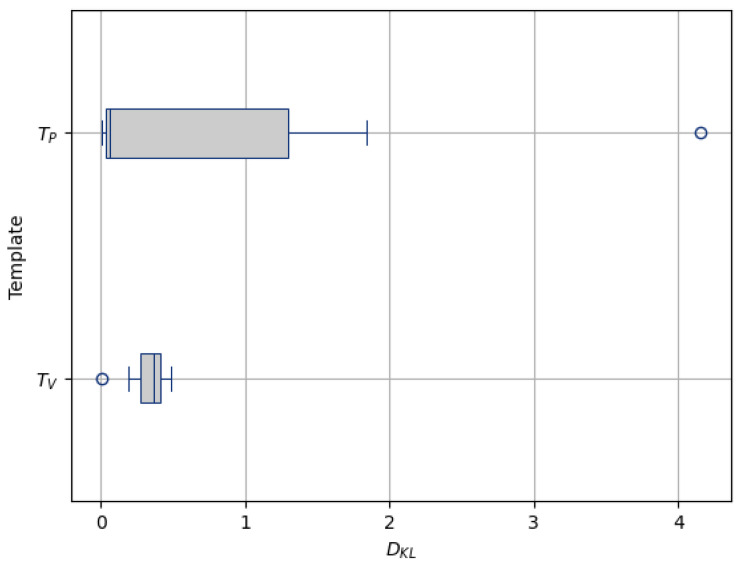
Distribution of $D_{KL}$ values for templates generated using patch-based estimation ($T_{P}$) and voxel-based averaging ($T_{V}$). Each gray-filled box with blue edges represents the interquartile range of the data, with the blue line inside indicating the median $D_{KL}$ value. Small circles denote outliers from the distribution.

- Sharpness
  Figure 14 visualizes the gradient magnitude for $T_{P}$ and $T_{V}$, with their corresponding AGM values summarized in Table 4. The AGM value for $T_{P}$ (60.958) was higher than that of $T_{V}$ (55.175). The higher AGM value for $T_{P}$ indicates that the patch-based approach resulted in a template with sharper edges compared with the voxel-based averaging method. This finding suggests that patch-based estimation of template intensity can lead to sharper templates compared with traditional voxel-based averaging.
- Contrast
  Table 4 presents the NMC values calculated for the pure WM and GM regions of the templates generated using the patch-based ($T_{P}$) and voxel-based ($T_{V}$) methods. Figure 15 visualizes these pure tissue regions in both templates. As shown in the table, the NMC value for $T_{P}$ (0.418) is higher than that of $T_{V}$ (0.393), indicating higher contrast in the former. This suggests that the patch-based approach yields a template with enhanced contrast between these tissues compared with the voxel-based averaging method.
- Robustness to Outliers
  Figure 16 shows the distribution of the $D_{KL}$ values calculated for each template ($T_{P}$ and $T_{V}$) and the population images, with their median values summarized in Table 4. The median $D_{KL}$ value for $T_{P}$ (0.057) is less than that of $T_{V}$ (0.368). Also, the $D_{KL}$ value for the introduced outlier image is 4.159 with $T_{P}$, while it is 0.001 with $T_{V}$, indicating that the former intensity is more similar to the population and is less influenced by outliers. This finding suggests that patch-based estimation of template intensity results in a template that more accurately reflects the most common intensity values in the population and is less sensitive to outliers compared with traditional voxel-based averaging.

### 4.3. Usability of Saudi Brain Template

**Figure 17:**
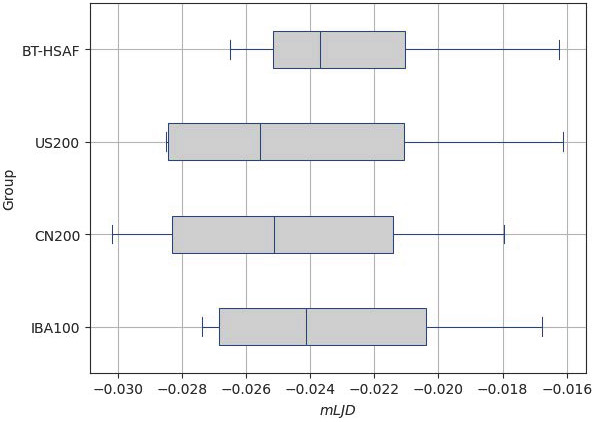
Distribution of mLJD values for the registered healthy Saudi adult female brain images to the four standard spaces: BT-HSAF, US200, CN200, and IBA100. Each gray-filled box with blue edges represents the interquartile range of the data, while the blue line inside indicates the median mLJD value.

Figure 17 shows the distribution of the mLJD values calculated from the registration of healthy Saudi adult female brain images to the four standard spaces: BT-HSAF, US200, CN200, and IBA100, with their median values summarized in Table 5. The median mLJD value for BT-HSAF (−0.02368) is the closest to zero, followed by IBA100 (−0.02413), CN200 (−0.02513), and US200 (−0.02557). This indicates that registering healthy Saudi adult female subjects to the BT-HSAF template results in the least volume changes compared with the other templates. These findings highlight the importance of using a population-specific brain template when registering the healthy Saudi adult female subjects, as it is more similar to the population and can preserve anatomical volumes.

## 5. Discussion

This study aimed to construct a representative brain template for healthy Saudi adult females using a homogeneous subset of T1-weighted MRI scans. We addressed challenges related to variability in raw data, scanner artifacts, and irrelevant anatomical structures through a series of preprocessing steps. Our template construction methodology integrates techniques designed to produce an unbiased, sharp, and high-contrast template that is robust to outliers and computationally efficient. Furthermore, we compare key evaluation aspects of our approach with previous studies, as summarized in Table 6 and discussed below.

Our evaluation of unbiasedness, measured using voxel-wise WD, demonstrated that the weighted template ($I_{wc}$) exhibited lower total and average WD compared with the unweighted template ($I_{c}$). This suggests that the use of similarity weighting in our method effectively mitigates bias during template construction. This finding is consistent with the work of Parvathaneni et al. [47], who also found that weighted templates, derived from scan-rescan reproducibility datasets, yielded more stable and less biased results. Their evaluation was conducted using distance metrics such as mean square error (MSE) and average relative distance (ARD) on cortical surface averages, which also showed improved stability with weighted approaches. These results reinforce the importance of population-specific weighting, a principle central to our study.

In terms of image quality, our patch-based template ($T_{P}$) demonstrated superior image quality compared with the voxel-based template ($T_{V}$) across several key metrics. It achieved a higher AGM, a higher NMC, and a lower $D_{KL}$ distribution. These metrics indicate that the patch-based method produces sharper images with better tissue contrast while being less sensitive to outliers. Coupé et al. [37] conducted a similar comparison between patch-based and voxel-based templates and found that the former offered superior contrast (with a higher NMC) and was less sensitive to outliers (with a lower $D_{KL}$), further supporting the robustness of our approach. Additionally, Yang et al. [44] applied a patch-based method for diffusion MRI and reported significant improvements in fiber orientation distributions, peak signal-to-noise ratio (PSNR), and artifact reduction. These findings suggest that our patch-based template construction method aligns well with the successes observed in these two studies, further validating its potential for producing high-quality, robust templates.

Regarding usability, we found that our BT-HSAF exhibited the closest *mLJD* distribution to zero, indicating that it required the least deformation during registration compared with IBA100, CN200, and US200. This result suggests that the BT-HSAF is highly compatible with the healthy Saudi adult female population, ensuring accurate alignment during image registration. These findings align with the research by Sivaswamy et al. [20], which demonstrated that the IBA100 template minimized deformation and improved segmentation accuracy when applied to Indian subjects. Additionally, Yang et al. [18] found that using population-matched templates significantly reduced registration deformation and enhanced segmentation accuracy. Their study also highlighted the morphological differences between ethnic groups and genders, further emphasizing the importance of developing templates that are specific to the population being studied. This reinforces the need for developing Saudi-specific brain templates tailored to different population subsets, which is critical for preserving anatomical features and improving the reliability of neuroimaging analyses.

We enhanced the computational efficiency of our approach by leveraging the parallel processing capabilities of Google Colaboratory [57]. All preprocessing, construction, and evaluation steps were parallelized, which reduced the total computational time from *X* to approximately $\frac{X}{C}$, where *C* represents the number of available processing cores. For instance, in the SMC method, the number of pairwise inter-subject registrations decreased from 2(N−1) to $\frac{2(N-1)}{C}$. Additionally, we implemented vectorization in place of nested loops, particularly in the patch-based estimation, and excluded zero-valued voxels from calculations. These strategies significantly improved processing efficiency. While no formal timing benchmarks were recorded, we observed noticeably faster and more scalable computations as a result.

While this study provides valuable insights, it also has several limitations:

- The current template was constructed using only one subset of the Saudi population (Section 3.1), and templates for other subsets were not developed.
- The sample size for this subset is relatively small, which limits the generalizability of the resulting brain template despite the dataset’s homogeneity and restricts the potential for meaningful statistical comparisons.
- Linear characteristics of the subset (e.g., brain length, width, and height) were not addressed; these were normalized through affine spatial normalization (Section 3.2.1), while the focus remained on nonlinear anatomical details solely (Section 3.3.2).
- The similarity weighting step assigned a single weight per brain image (Section 3.3.1), applied uniformly across all voxels.
- In the template intensity estimation step (Section 3.3.3), all patches were included in each iteration without selective filtering.

Building on the findings and limitations of this study, the following research directions are recommended for further exploration:

- Constructing multiple brain templates for a broader range of Saudi population subsets, using sufficiently large sample sizes and accounting for variations in gender, age groups, and pathological conditions.
- Incorporating multiple imaging modalities (e.g., T2-weighted MRI, CT, fMRI, PET, DTI) to enhance both anatomical and functional relevance of the templates.
- Developing a comprehensive Saudi brain atlas that includes tissue probability maps and region labeling alongside various types of brain and head templates, providing a richer resource for neuroimaging studies.
- Integrating the developed atlas into widely used neuroimaging tools such as FreeSurfer [95], FSL [61,62,63], and SPM [96] to facilitate adoption in research and clinical workflows in Saudi Arabia. This integration could support automated segmentation, early abnormality detection, and treatment or surgical planning, particularly as advanced neuroimaging protocols become more common in clinical practice [97].
- Replacing affine spatial normalization with rigid registration and directly incorporating linear anatomical characteristics into the template construction process to yield more representative templates.
- Using localized similarity weights (rather than a single global weight per image) to improve structural unbiasedness.
- Implementing early discarding of mismatched patches, as proposed by Coupé et al. [91], to reduce computational costs and improve robustness.
- Exploring the effects of different patch sizes on the quality of the constructed template.

## 6. Conclusions

This study introduced an integrated approach for constructing a representative and unbiased brain template specifically tailored to the healthy Saudi adult female population. By integrating several key techniques, we addressed critical challenges in template creation. Specifically, we combined the SMC method with a covariance-based weighting scheme to mitigate bias arising from dataset asymmetry and over-represented brain structures. Furthermore, we incorporated patch-based intensity estimation, which ensured high image quality, yielding a sharp, high-contrast template robust to outliers. Crucially, we used a homogeneous subset of MRI scans from Saudi subjects—a first for this population—which allowed us to create a template expected to be more representative and effective for registration purposes compared with non-population-specific templates. This newly developed brain template for Saudi adult females represents a valuable resource for future neuroimaging studies focused on this population, promising to improve the accuracy and reliability of anatomical analyses and contribute to a deeper understanding of brain structure and function in Saudi individuals.

## Figures and Tables

**Table 2:** Image similarity weights as prior knowledge for the template construction.

Image	Weight
1	0.143001
2	0.143001
3	0.138172
4	0.145285
5	0.135802
6	0.147039
7	0.147702

**Table 3:** Comparison of WD for $I_{wc}$ and $I_{c}$ (sum and average).

Template	Sum	Average
$I_{wc}$	33,566,831.060	3.935
$I_{c}$	33,735,950.577	3.955

**Table 4:** Comparison of the intensity quality for $T_{P}$ and $T_{V}$ (AGM, NMC, and $D_{KL}$).

Template	AGM	NMC	$D_{\mathrm{KL}}$†
$T_{P}$	60.958	0.418	0.057
$T_{V}$	55.175	0.393	0.368

† The median value of the $D_{KL}$ results.

**Table 5:** Median mLJD values for the healthy Saudi adult female brain images registered to different template spaces (BT-HSAF, US200, CN200, and IBA100).

Template	mLJD
BT-HSAF	−0.02368
US200	−0.02557
CN200	−0.02513
IBA100	−0.02413

**Table 6:** Comparison of evaluation aspects between the current study and previous work.

Evaluation Aspect	Current Study	Previous Work
Unbiasedness	Used voxel-wise WD Weighted template ($I_{wc}$) showed lower total and average WD than unweighted $I_{c}$	Parvathaneni et al. [47]: Assessed using scan-rescan datasets and distance metrics (MSE, ARD) Weighted cortical averages reduced bias and improved stability
Image Quality	Patch-based template ($T_{P}$) outperformed voxel-based ($T_{V}$) Higher AGM, higher NMC, lower $D_{KL}$ for $T_{P}$	Coupé et al. [37]: Patch-based templates had better contrast and less sensitivity to outliers Yang et al. [44]: Cleaner fiber orientation distributions, improved PSNR, and fewer artifacts
Usability	BT-HSAF had mLJD closest to zero Compared with IBA100, CN200, US200	Sivaswamy et al. [20]: IBA100 improved deformation and segmentation for Indian subjects Yang et al. [18]: Population-matched templates reduced registration deformation and enhanced segmentation accuracy across ethnic/gender groups

## Data Availability

The data presented in this study is not publicly available due to privacy.

## List of Symbols

*S*	Raw scans
*I*	Preprocessed images set
*N*	Number of images
$I_{j}$	Random image from the set *I*
$I^{\prime }$	Aligned *I*
$I_{wc}$	Weighted center image
$I_{c}$	Unweighted center image
*T*	Template
*t*	Iteration counter
$T^{t}$	Template at iteration *t*
Δ	Difference between $T^{t}$ and $T^{t-1}$
$T_{P}$	Template from patch-based estimation
$T_{V}$	Template from voxel-based averaging
*F*	Features
PC	Principal components
Σ	Covariance matrix
$\Sigma^{-1}$	Inverse of Σ
*W*	Image similarity weights
NW	Normalized image similarity weights
$d_{ji}$	Displacement from $I_{j}$ to each $I_{i}\in I:i\neq j$
$d_{wj}$	Weighted displacement for $I_{j}$ to reach $I_{wc}$
$d_{ic}$	Displacement from $I_{i}\in I$ to center image
$P_{T}$	Set of template patches
$P_{I}$	Set of $I^{\prime }$ patches
*D*	Euclidean distances
*h*	Gaussian bandwidths
*w*	$I^{\prime }$ nonzero voxel weights
nw	$I^{\prime }$ nonzero voxel normalized weights
*V*	Set of nonzero voxel indices
*K*	Number of nonzero voxel indices
*v*	Voxel index
*	Element-wise multiplication
WD	Weighted Displacement
AGM	Average Gradient Magnitude
NMC	Normalized Michelson Contrast
$D_{KL}$	Kullback-Leibler Divergence
$WM_{\max}$	Maximum intensity in WM
$GM_{\min}$	Minimum intensity in GM
${∥.∥}_{2}$	The L2-norm
∣·∣	The absolute value
*x*	Intensity or voxel value
*M*	Number of template voxels
P(x)	Probability of *x* in template distribution
Q(x)	Probability of *x* in $I^{\prime }$ distribution
$J_{v}$	Jacobian matrix at *v*
$\det(J_{v})$	Determinant of $J_{v}$
*∇*	Gradient operator
*X*	Total computational time
*C*	Processing cores
